# Use of a convolutional neural network for direct detection of acid-fast bacilli from clinical specimens

**DOI:** 10.1128/spectrum.00602-25

**Published:** 2025-06-23

**Authors:** Paul English, Muir J. Morrison, Blaine Mathison, Elizabeth Enrico, Ryan Shean, Brendan O'Fallon, Deven Rupp, Katie Knight, Alexandra Rangel, Jeffrey Gilivary, Amanda Vance, Haleina Hatch, Leo Lin, David P. Ng, Salika M. Shakir

**Affiliations:** 1 ARUP Institute for Research and Innovation in Diagnostic and Precision Medicine, ARUP Laboratories 33294, Salt Lake City, Utah, USA; 2 ARUP Technical Operations Infectious Diseases, ARUP Laboratories 33294, Salt Lake City, Utah, USA; 3 Department of Pathology, University of Utah 161530, Salt Lake City, Utah, USA; University of Cincinnati, Cincinnati, Ohio, USA

**Keywords:** acid-fast bacilli, mycobacteria, artificial intelligence, digital pathology, microbiology, object detection, tuberculosis, *Mycobacterium*

## Abstract

Mycobacteria, including *Mycobacterium tuberculosis* (MTB) and non-tuberculosis mycobacteria (NTM), are important causes of infectious disease and cause significant mortality and morbidity globally. Fast detection is extremely important to reduce transmission and mortality associated with these infectious agents. Manual smear microscopy is a cost-effective tool for diagnosing and monitoring of these organisms; however, it is labor-intensive and requires highly-trained personnel. We present the development of an artificial intelligence computer vision process using a deep convolutional neural network to detect acid-fast bacilli (AFB) from Kinyoun acid-fast stained slides. We collected 231 clinical specimens between August 2023 and June 2024. Following acid-fast staining, whole slide images (WSI) were digitized, and AFB organisms were manually annotated. A machine learning computer vision model was trained using 11,411 annotated organisms across 109 WSI. Model predictions were correlated with final culture-confirmed results. The final model estimated AFB density per 1000 x microscope field of view (FOV). Using a density threshold of ≥10^−2^ AFB/1000xFOV (corresponding to 1 + per Clinical and Laboratory Standards Institute (CLSI) guideline M48) to predict positive culture results, the model correctly classified 68% of validation slides, with a sensitivity of 79% and specificity of 63%. Manual AO compared to final culture read showed sensitivity of 76% and specificity of 96%. Although performance of our model was not sufficient to be clinically implemented in our laboratory, our study provides a framework for AI-based AFB detection and a publicly available data set to support future advancements in automated detection of AFB.

**IMPORTANCE:**

We present the development of an artificial intelligence model to detect acid-fast bacilli (AFB) directly from stained clinical smears. While the model’s current performance requires further improvement to be clinically useful in our lab, we detail our approach and share our expertly annotated data set to support future advancements in this area. By building on our work, researchers can develop better algorithms to improve the diagnosis of AFB, reducing the burden on laboratory staff and improving diagnostic speed and accuracy of these medically important organisms.

## INTRODUCTION

Mycobacteria are an important cause of infectious disease and cause significant mortality and morbidity globally. A large burden of this is caused by *Mycobacterium tuberculosis* (MTB), which was estimated to kill 1.6 million people in 2021 (1) and 1.3 million people in 2023 (2). As tuberculosis (TB) is preventable, treatable, and curable, fast and accurate detection of AFB on a smear is extremely important to reduce transmission, provide a preliminary diagnosis, and reduce mortality.

*Mycobacterium* species are aerobic, acid-fast, non-motile bacilli. *Mycobacterium,* including MTB, bacilli are extremely small (~2-4 μm × 0.2-0.5 μm), and detection of even a single organism per slide can be diagnostically relevant (3). Although MTB can infect a variety of organ systems and be seen on histopathological tissue sections (4), the lungs are the most common site of infection (5). While molecular techniques are available to detect and diagnose MTB (6), they are expensive and can remain positive even when the patient is clinically responding to effective treatment. Direct microscopic examination offers a relatively inexpensive and reliable method for detecting AFB and assessing treatment effectiveness. Additionally, presence of visible AFB on clinical sputum smears correlates with infectivity (7) making manual microscopy effective at assessing transmission risk and need for airborne isolation precautions. Despite advancements in molecular diagnostics, culture remains the gold standard for MTB diagnosis and direct detection of AFB on smears continues to be a mainstay of diagnostic procedures. The World Health Organization (WHO) recommends microscopy of acid-fast stained smears as the first-line approach for diagnosing pulmonary TB. However, the sensitivity of smear microscopy for detection of pulmonary TB is only 50-60% compared to culture (8).

The Ziehl-Neelsen (ZN) method is often used to stain and identify AFB directly from smears for evaluation under bright-field microscopy (9). Additionally, modified staining techniques, such as the Kinyoun or Fite stains, can also be used to visualize AFB under brightfield microscopy. Fluorescent microscopy, which allows easier detection of AFB during manual microscopy, can be performed with fluorescent stains such as Auramine O (AO), alone or in combination with rhodamine. Because fluorescent stains make detection of AFB significantly easier, increasing sensitivity of microscopy (10), and reducing screening time, the College of American Pathologist (CAP) and Centers for Disease Control and Prevention (CDC) recommend them as the primary method for acid-fast staining clinical specimens (CAP Microbiology Checklist: MIC.32100).

However, even with fluorescent stains, manual microscopy is a repetitive and time-consuming task that requires highly trained laboratory staff. Additionally, it is a CAP requirement that labs must report acid fast stain results within 24 hours of specimen receipt (CAP Microbiology Checklist: MIC.31200). This turn-around-time (TAT) requirement combined with variable day-to-day specimen volumes makes adequate and efficient staffing extremely difficult. Furthermore, many local US laboratories have consolidated or closed AFB testing and send testing to reference laboratories – causing volumes for laboratories that still perform this testing to rapidly increase.

Due to the long-standing difficulty in the detection of AFB by manual microscopy, as well as recent advancements in whole slide imaging (WSI), computer vision, and artificial intelligence (AI), there has been much interest in developing an automatic method for the detection of AFB (11). If an AI-based process could show increased sensitivity and efficiency compared to fluorescent microscopy, it could supplant fluorescence as the gold standard for AFB detection. In the last decade, there has been a significant effort to develop AI image analysis to assist with AFB detection. As fluorescent microscopy is generally considered easier for human technologists and is a regulatory requirement, there has also been interest in developing automatic image analysis of fluorescently stained slides. Several devices have been developed and studied, showing variable sensitivity but potential for reductions in manual microscopy workloads (12–14). Some recent studies have shown relatively high sensitivity but low specificity (15). There are many difficulties with automatic image acquisition and analysis for fluorescent microscopy, including autofluorescence, focusing on problems producing substandard images or even total scan failures (16). Additionally, the cost of instruments is prohibitive for routine use, and the time to scan a fluorescent slide is significantly increased compared to that for conventional light microscopy scanners (17). As a result, there has been much interest in building automatic pipelines and image analysis tools for the detection of AFB with light microscopy and traditional carbol fuchsin stains. There have been several recent studies showing favorable sensitivity compared to human microscopy and also potential personnel time savings (18–20). ZN staining has also been studied for automatic detection of *Mycobacterium* in histopathological tissue sections (21).

**Fig 1:**
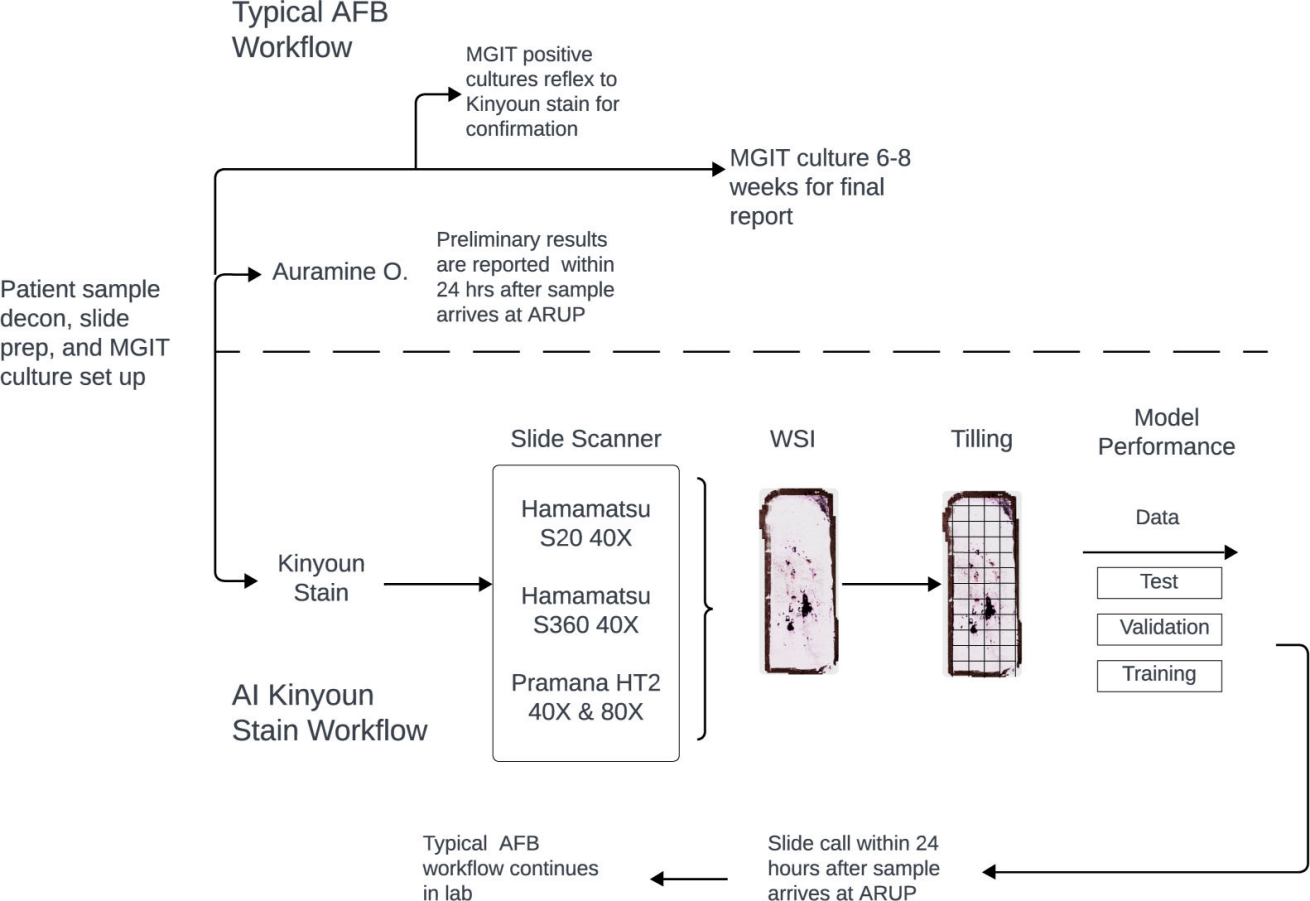
Summary of study workflow including routine clinical operations as well as additional study procedures. WSI: whole slide images.

To address the difficulties associated with manual microscopy and automatic fluorescence scanning, our laboratory developed a process for automated identification of AFB direct from Kinyoun-stained clinical specimens, including slide staining, scanning, and a computer vision approach to detect possible AFB (Fig. 1).

## MATERIALS AND METHODS

### Clinical slide set

During routine operations at our United States based national reference laboratory between August 2023 and June 2024 a total of 231 specimens sent for AFB testing were collected (Table 1). Initially AO +specimens were randomly selected for expert annotation (see below), and later AO- specimens were randomly selected for additional training diversity and to build the validation set. Specimens came from a variety of sources including sputum, bronchoalveolar lavage (BAL), aspirates, wounds, tissues, body fluids, and isolates sent for AFB identification.

**TABLE 1:** Distribution of sample types for clinical samples

Source	No. of specimens	AO positive	AO negative	MGIT culture positive	MGIT culture negative
Sputum	68	38	30	42	27
Respiratory other	68	14	54	18	50
Tissue	37	1	36	0	37
Body fluid	34	3	31	3	31
Wound	17	0	17	1	16
Not specified/other	7^*a*^	3	2	5	2

^*a*^ AO not performed in two cases (direct isolate and stool).

Clinical specimens were processed according to our laboratory standard operating procedures. Briefly, specimens were digested and decontaminated using a liquefying agent (N-acetyl-L-cysteine or NALC) and a decontaminating reagent (NaOH with sodium citrate) in a 1:1 ratio. The mixture is allowed to contact the specimen for 15–20 minutes, with vortexing until the specimen has been liquefied. Phosphate buffer was then added to neutralize NaOH before centrifugation at 3,100 × *g* for 22 mins at 18°C. After centrifugation, the supernatant was discarded, and the pellet was resuspended in phosphate buffer. This specimen was used to inoculate Mycobacteria Growth Indicator Tubes (MGIT, Becton Dickinson, Franklin Lakes, USA) (with growth supplement) and Middlebrook 7H11 selective agar (Hardy Diagnostics, Santa Maria, USA) and to prepare slides for direct stains. For auramine O and Kinyoun stains, one drop of 0.2% bovine serum albumin in saline was added to affix the specimen to the slide. Slides were heat-fixed at 80°C−90°C for 30 minutes before staining.

The residual sample material not used for clinical testing was used to create additional slides for training and testing of our computer vision model. All final culture results were reported and correlated with the original smears (Table 2).

**TABLE 2:** Microbial species identification in positive samples by sample type^*a*^

Source	No. of positives	Organism						
		MTBC	MAC	MABS	MKC	*M. simiae*	*M. cosmeticum*	Unable to ID
Sputum	42	13	22	4	1	1	–^*c*^	1^*b*^
Respiratory other	18	1	12	3	1	–	1	–
Tissue	0	–	–	–	–	–	–	–
Body fluid	3	3	–	–	–	–	–	–
Wound	1	–	–	1	–	–	–	–
Not specified/other	4	1	3	–	–	–	–	–

^*a*^ MTBC, *Mycobacterium tuberculosis* complex; MAC, *Mycobacterium avium* complex; MABS, *Mycobacterium abscessus*; MKC, *Mycobacterium kansasii* complex.

^*b*^ The organism grew on culture but could not be identified by molecular techniques.

^*c*^ –, not identified.

### Slide scanning

An initial scanner comparison study was performed using the NanoZoomer S360 (Hamamatsu Photonics, Hamamatsu City, Japan), NanoZoomer S20 (Hamamatsu), Infinity (Motic Digital Pathology, Emeryvill, USA), VS200 (Evident Scientific, Tokyo, Japan), and HT-2 (Pramana, Cambridge, USA) scanners.

For the NanoZoomer S360, NanoZoomer S20, Infinity, and VS200, the scan area was manually selected, and slides were scanned with default scan settings using a single Z-layer. The HT-2 scanner with default settings performed volumetric scanning with algorithm based automatic detection of the scan area.

The NanoZoomer S360, NanoZoomer S20, and HT-2 were identified as the most successful at scanning the AFB slides and were chosen for further algorithm development. The HT-2 scanner was configured to its standard volumetric settings, with nine Z-layers at 1.25 µm intervals. The NanoZoomer S360 and The NanoZoomer S20 scanners were originally configured to scan 1z layer per default settings. Later, the NanoZoomer S360 was configured to match the settings used on the Pramana as closely as possible, scanning nine Z-layers at 1.3 µm intervals. Scanner settings and performance characteristics are summarized in Table S1.

All slides were scanned at 40 x magnification (Hamamatsu, 0.23 microns per pixel; Pramana Scanner 1 (40x objective), 0.247 microns per pixel; Pramana Scanner 2 (20x objective with doubler; 0.288 microns per pixel). Additionally, slides were also scanned on the HT-2 at 80 x total magnification to evaluate potential improvements in AI development. However, the increased scan time at 80 x was deemed prohibitive, and all further development used 40 x scans.

### Data set creation

Training and validation data sets were created by randomly sampling specimens and stratifying by sample type, source, AFB-positive classification (positive or negative), and grade (negative, 1+, 2+, 3+, and 4 + according to CLSI M48) (22). To avoid data leakage between training and validation data sets, all WSI scans of a given physical glass slide were placed in the same data set. (Table S2) summarizes the data set splits. Each specimen could have multiple glass slides created from it, and each glass slide could have multiple WSI scans and tiles created from it.

Both the object detection training and validation data sets included tiles created on the HT-2, NanoZoomer S20, and NanoZoomer S360 scanners. For the validation set assessing WSI-level prediction performance, only WSI from HT-2 was included. Due to operational reasons, the targeted clinical implementation would exclusively be on the Pramana HT-2 scanner. Additionally, 117 new WSI scanned on the HT-2 from 53 specimens were added to this validation set. These added specimens were not part of the object-level validation set because they had no object-level annotations.

### Image extraction and standardization

Portable network graphics (.PNG) images were extracted from each scanned WSI across a consistent grid of small tiles. To ensure that every organism in its entirety would be included in at least one tile, the tile grid was constructed such that neighboring tiles were partially overlapping with an overlap larger than the size of a typical AFB. This approach also increased training data diversity by showing the model the same annotated objects with partially different backgrounds.

To standardize physical size of objects from a variety of scan magnifications, all tiles were resized to 256 × 256 pixels at a consistent resolution of 0.2878 microns per pixel (MPP). For example, 80 x scans with an original resolution of 0.1542 MPP were processed by extracting 478 × 478 pixel tiles and resizing to 256 × 256 pixels. This ensured that each tile covered a uniform physical area of ~74×74 µm with even resolution regardless of original scan magnification, and that the scale of objects was standardized. The reduction in resolution of the 80 x scans was deemed to be acceptable as 80 x scans proved too time-consuming to be used in our planned clinical implementation.

### Manual AFB annotation

Bounding box annotations were created from pre-extracted tiles by trained and certified AFB laboratory staff proficient in performing AFB smear microscopy in our CLIA- and CAP-certified clinical laboratory. Annotations were generated using Label Studio (23), with additional annotations generated within the WSI viewing software (Digital Slide Archive (24). Objects were labeled as either “AFB,” “mimic,” “non-AFB,” or “unknown”; however, almost all annotated objects were AFBs. Slides for annotation were preferentially selected from AO-positive slides to enrich for objects to annotate.

These annotations were then combined and converted to a consistent format containing the object class, location coordinates in the WSI, and associated metadata such as timestamp and annotator. In total, the data set contains 11,411 unique manually annotated organisms across 109 WSI from 68 specimens. However, due to real-life variability in specimen positivity and density (by definition, a 4 + specimen has 100–1,000x more organisms than a 1+), most AFB object annotations came from a small fraction of the most densely populated slides. For example, a single slide accounted for 7,197 annotations, with an additional 2,813 annotations from only four slides.

### Tile extraction and class balancing

To select tiles for the WSI-classification validation set, 10,000 tiles were randomly sampled from a tile grid covering a 1 cm^2^ region near the center of each scan away from the wax ring for a total area of approximately 50 mm^2^, roughly 4 x the minimum area (13 mm^2^) recommended by CLSI for AO-stained AFB smear examination (22). Tiles near the center of the scan were chosen because preliminary experiments indicated whole WSI sampling reduced performance (possibly due to increased noise at the edge of smears or near the wax ring), and most annotations were from the center of the scan. Tile selection for object-level detection splits required a more nuanced approach. For AFB-positive specimens, only tiles containing annotated objects were extracted. For AFB-negative specimens, tiles were originally extracted with a random subset of tiles from a 1 cm^2^ region near the center of the scan. To help reduce false-positive predictions in subsequent algorithm re-training iterations, tiles from AFB-negative WSI were selected only if they contained predictions from a previously trained model as these predictions represented false positives.

While general machine learning best practice suggests matching class imbalance (the ratio of positive to negative samples in the training set) to approximate the expected real-world class imbalance, routine clinical use would be expected to have an extreme imbalance in negative to positive tiles (>100:1 and potentially >1,000:1). In initial experiments, these ratios led to poor model performance, with training usually collapsing and producing no predictions. Therefore, the manually set imbalance ratio of negative to positive prelabeled tiles in the training set was reduced to 10:1–3:1, which improved training stability and downstream performance.

### Algorithm and model development

Conceptually, the WSI classifier can be viewed in two stages: i) a deep learning object detection model to detect individual AFB; ii) an aggregation operation that takes the set of predicted AFB across a WSI and outputs an AFB + or AFB– decision for the entire WSI.

#### Object detection training

Object detection began with a publicly available off-the-shelf model architecture (FCOS (25) with standard ConvNeXt (26) backbone pretrained on ImageNet1k, implemented with torchvision (27), which was then fine-tuned on tile data sets extracted from our WSIs. Although Faster R-CNN (28) and ResNet (29) were investigated, preliminary testing indicated FCOS +ConvNext performed best and was selected. As described above, to reduce the number of false-positive predictions, we used a two-step training approach. In the first step, the model was trained on 15,353 tiles including 3,683 tiles positively annotated as AFB and 11,670 tiles randomly sampled from negative WSIs. Then, any tile originally from a negative WSI that was predicted to contain an AFB was assumed to be a false-positive. Next, a second-round model was trained using 48,193 tiles, including 11,414 positive AFB tiles and 36,779 tiles from negative WSIs that overlapped with a false-positive prediction from the first-round model.

#### Object detection validation

The first-round validation set contained 6,330 tiles, including 420 containing AFB object annotations, while the rest were randomly sampled negatives. The second round-validation set contained 5,072 tiles, including 408 AFB positive tiles, and the remainder were negative tiles that overlapped with false-positive detections from the first round.

#### Whole slide predictions

Since each WSI is divided into overlapping tiles, the total number of detected objects could be falsely increased due to double-counting objects in overlapping tiles. Therefore, for each WSI, all predictions across all tiles were converted to WSI-global coordinates and non-maximum suppression was applied to all detections across the WSI at once. This largely removes multiple detection events.

For aggregating object level detections into WSI-level classification, a simple decision rule based on thresholding the estimated AFB density was used. Estimated AFB density was computed as the total number of AFB objects detected by the model divided by the total area of tiles for that WSI.

## RESULTS

### Algorithm performance

#### Model evaluation and selection

**Fig 2:**
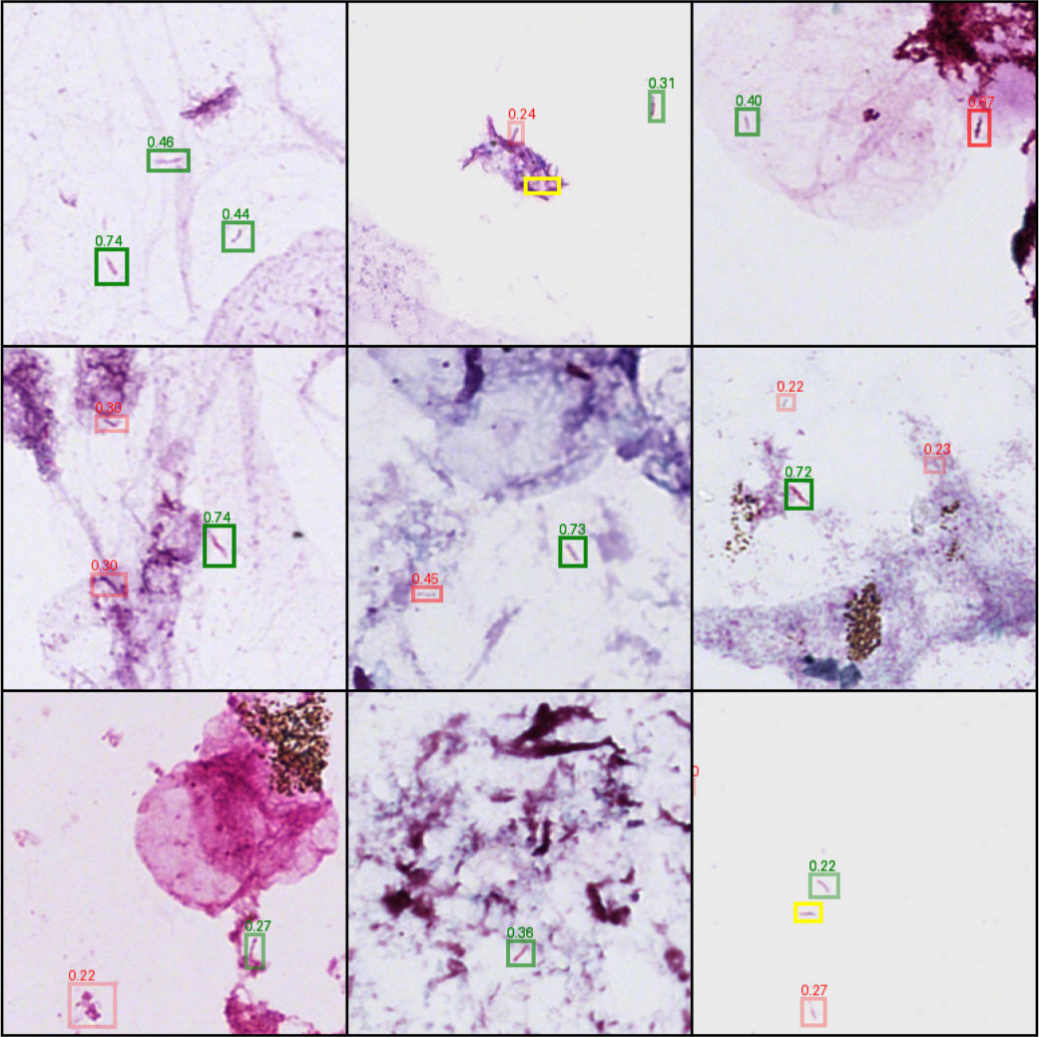
Example predictions across a curated sample of tiles. True positives are green, false positives are red, and false negatives are yellow. For true positives, we show only the model’s predicted bounding box and not the ground-truth annotated box to reduce visual clutter. The confidence score associated with each predicted box is shown, and the box transparency also indicates the confidence of the model’s predictions, with darker/fainter boxes corresponding to higher/lower confidence, respectively. The upper-middle and lower-right tiles are from HT-2 scans while the other seven are from NanoZoomer S360 scans. See Fig. S1 for a representative random sampling of tiles from our validation set.

**Fig 3:**
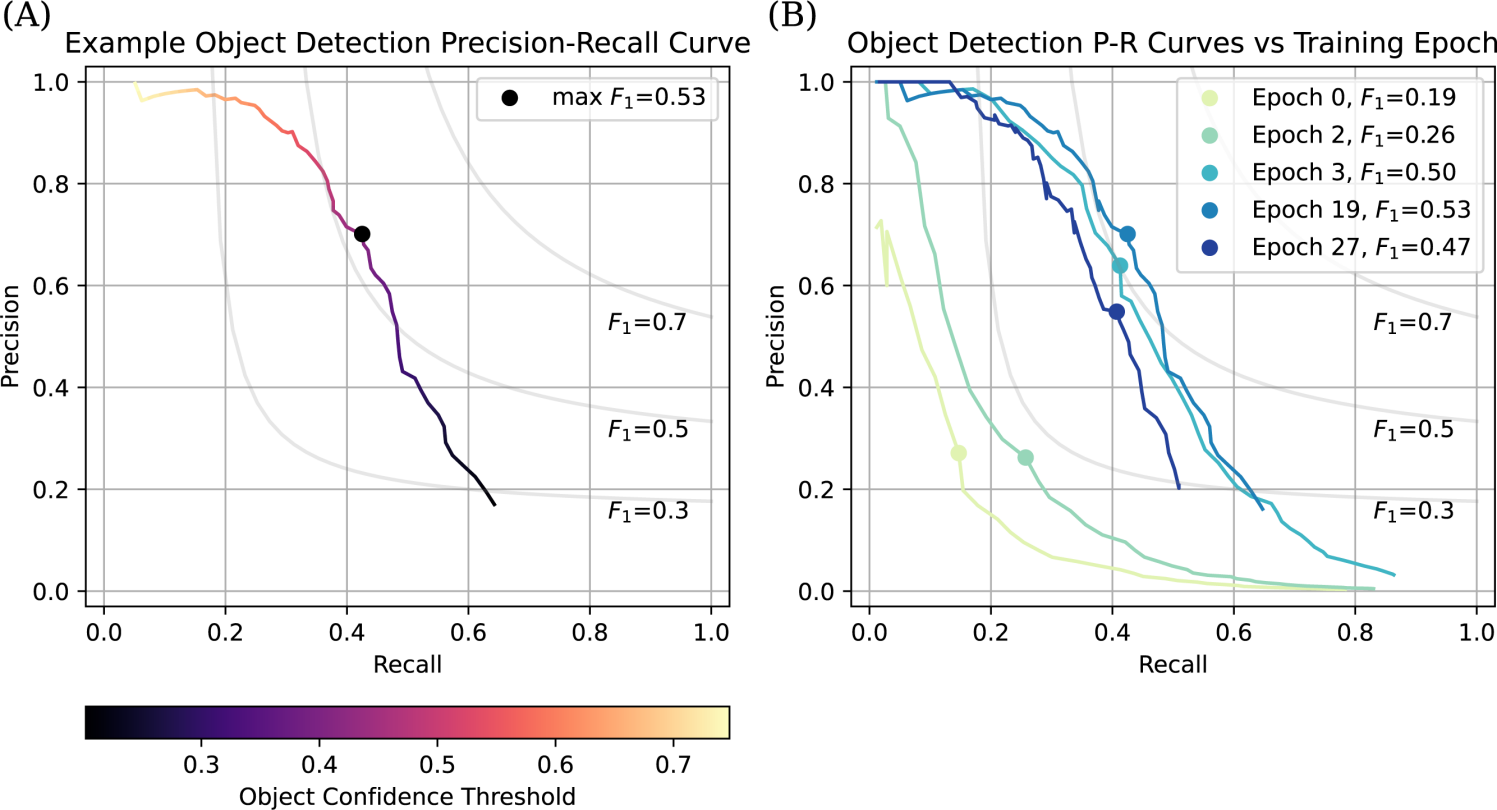
Example Object Detection Precision-Recall Curves. (**A**) Precision-recall tradeoff as object confidence score threshold varies. Each point represents model performance at a given object confidence threshold, shown by the color gradient. The x-axis corresponds to the recall at each given object score threshold. The y-axis represents the corresponding precision at each given object score threshold. With a very high object score threshold, precision is very high as only the most confident predictions are called AFB, and recall is very low as many true positives are missed. Conversely, at a low confidence threshold, recall is high as many true positives are caught, but precision is very low due to an increased number of false positives. Curved gray lines represent various precision and recall values that would give the corresponding F1 score. (**B**) Evolution of the model’s performance on the validation set as training epochs progress. The x-axis represents recall. The y-axis represents precision. Each line is color-coded by epoch number. The course of each line represents the various precision and recall scores as the object score threshold is changed across the range of confidence scores created by the model. For unknown reasons, as training progressed, the dynamic range of confidence scores decreased, which is shown by the different end points of each line at different training epochs. Curved gray lines represent the range of precision and recall values for the corresponding F1 score. Note the drop in performance at epoch 27 compared to epoch 19.

The object detection model outputs areas of the slide predicted to contain AFB, assigning each prediction a confidence score representing the model’s certainty (Fig. 2). The confidence threshold used to make the final determination if an object is an AFB directly impacts model performance, resulting in a tradeoff between precision (the proportion of predicted positives that are true positives) and recall (proportion of total true positives that are predicted) (Fig. 3A). The F1 score, a harmonic mean of precision and recall, provides a single value to compare these tradeoffs at a given confidence threshold.

Model development and training is a dynamic and iterative process with many tunable parameters. For each iteration, precision, recall, and F1 score were calculated for all possible confidence thresholds. To compare different model configurations and iterations, we used the maximum F1 score achieved as the primary metric, providing a single, standardized, repeatable, and interpretable value for comparison.

##### Training epochs

To optimize the number of training epochs (a single pass through the training data updating the model), each training epoch was followed by scoring the model’s predictions on the validation set.

Since the backbone was ImageNet-pretrained and the FCOS head was randomly initialized, we began by training only the FCOS head for three epochs while keeping the backbone parameters frozen. After this “warm-up” phase, the backbone was unfrozen, allowing all model parameters to train and be subsequently adjusted. With this approach model performance rapidly improved with each subsequent epoch (Fig. 3B). The maximum F1 score of 0.53 was achieved in epoch 19, after which overfitting began to reduce performance on the validation set. Therefore, we used the model checkpoint from epoch 19 for downstream analysis. For a detailed overview of all final model parameters, the entire model is available at https://huggingface.co/arup-ri/afb.

##### WSI predictions

For the final model, all predicted AFB produced by the object detection model were used to calculate the WSI AFB density (number of predicted AFB per 1,000 x FOV on the physical slide).

**Fig 4:**
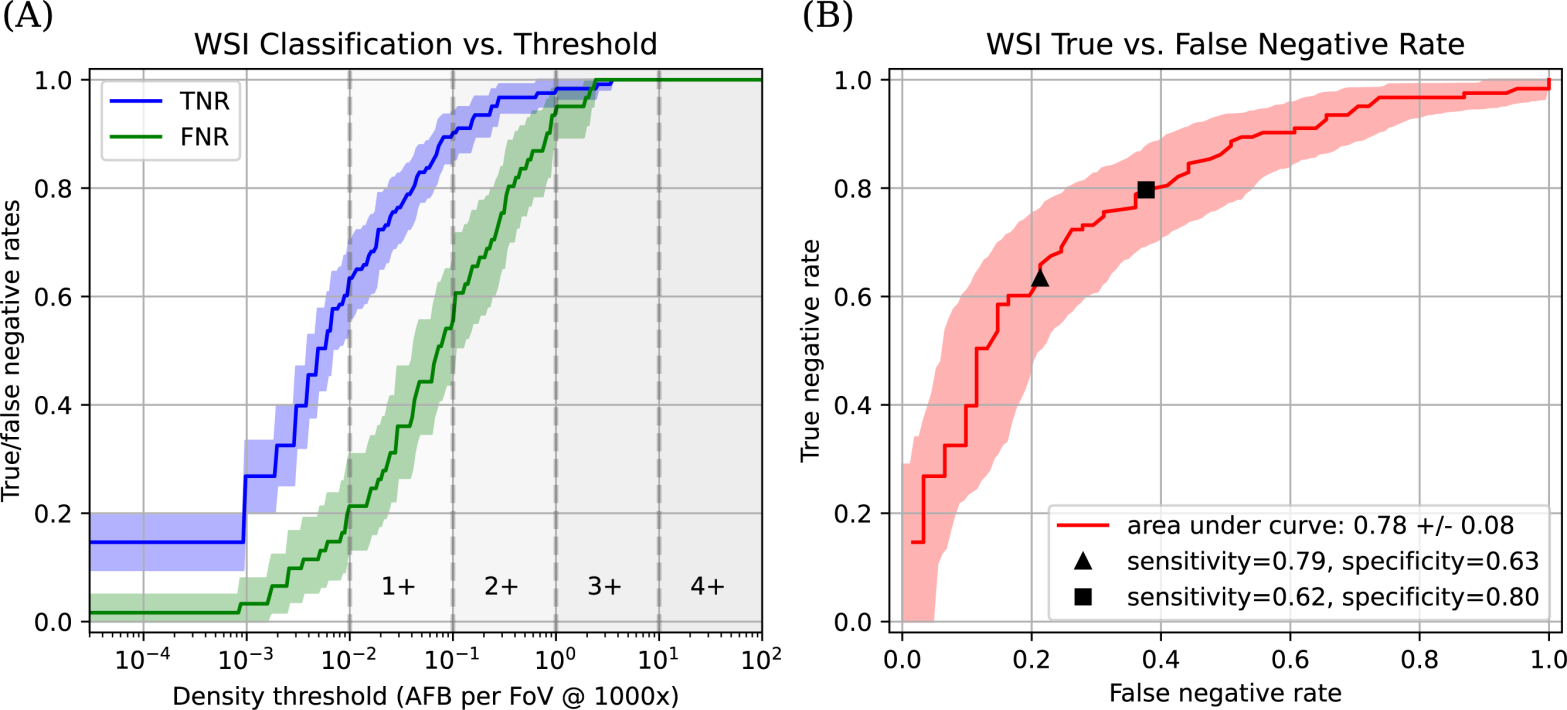
Model-predicted WSI-level classification results on our validation set. (**A**) True negative and false negative rates of AFB detection on the validation set as a function of predicted AFB density required to call a WSI positive for AFB. The density ranges for 1+, 2+, 3+, and 4 + grades on the CLSI M48 scale are shown as a point of comparison. Each point along the X-axis represents a different AFB density above which a WSI would be called positive/negative for AFB. The green line represents the false negative rate, with the percentage of false negatives expected on the validation set on the Y-axis. The blue line represents the true negative rate, with the percentage of true negatives on the Y-axis. Shaded confidence bands are nonparametric bootstrap resampling estimates of the 5th percentile and 95th percentile for the corresponding line. (**B**) True negative versus true positive rates as a function of the predicted AFB density threshold. As the density threshold changes, there is a tradeoff between true and false negative rates, shown by the path of the line. The x-axis shows false negative rate, and the y-axis shows a true negative rate. The triangle represents a decision threshold of 10^−2^ AFB/1000xFOV (M48 1+) with a corresponding sensitivity of 79% and specificity of 63% on our validation set. The square represents a decision threshold of 4´10^−2^ AFB/1000xFOV, which corresponds to a sensitivity of 62% and a specificity of 80%. The AUC can be interpreted similarly to the standard receiver operating curve. The shaded confidence band represents the nonparametric bootstrap resampling estimate of the 5th and 95th percentile estimates.

Full model performance varied by the predicted density of AFB used to call a slide positive or negative (Fig. 4A). Due to the intended clinical use case of using this model to screen out negatives, performance was evaluated using the true negative rate and false negative rate instead of the more common true positive vs false positive receiver operator curves. As the density threshold is changed, there is a necessary tradeoff between true negatives and false negatives (Fig. 4B).

Using an estimated AFB density of 10^−2^ AFB/1,000 x FOV (1+) or greater to predict the final culture result, there were correct predictions on 68% of slides in the validation set, with a sensitivity of 79% and specificity of 63%. With a different threshold of 4 × 10^−2^ AFB/1,000 x FOV, the model correctly predicted 74% of slides in the validation set, with a sensitivity and specificity for detection of AFB of 62% and 80%, respectively. The area under the curve (AUC) can be interpreted similarly to the standard area under the receiver operating characteristic, with a perfect classifier having an AUC = 1 and random guessing having an AUC = 0.5. The total AUC for the final model was 0.78 (SD 0.04).

### Manual AO performance

Additionally, manual AO reads were correlated with final culture results. Out of all 231 specimens, the AO read corresponded to the final culture read in 90% (208/231) of cases. Additionally, there were seven AO slides read as positive that ended up negative by culture and 14 negatives that ended up positive—corresponding to a sensitivity of 76% and specificity of 96%.

### AI prediction vs manual AO

Overall, the final validation set contained 105 specimens analyzed by the model. Each specimen could have multiple WSIs; if the model predicted positive AFB at a decision threshold set to M48 1+, on *any* WSI, the specimen was counted as AI-positive. Across all 105 specimens, human AO reads were negative 82 times and positive 23 times, while the AI prediction was negative 50 times and positive 55 times. The overall sensitivity of the AI prediction compared to human AO reads was 83%, and the specificity was 56%.

When splitting the WSI by the specimen source, there were 28 sputum specimens in the validation set. Human AO reads were negative 15 times and positive 13 times, whereas AI prediction showed 6 negatives and 22 positives. The sensitivity of the AI model compared to human AO for sputum samples was 92% with a specificity of 33%.

For the remaining 77 specimens (all other specimen types), human AO reads were negative 67 times and positive 10 times, while AI prediction showed 44 negatives and 33 positives. For non-sputum specimen types, AI prediction compared to human AO reads showed a sensitivity of 70% and specificity of 61%.

## DISCUSSION

Overall, we present a machine learning approach to detect AFB from Kinyoun-stained direct patient smears using bright-field WSI. Additionally, we provide our models and annotated data sets. As a clinical reference lab, the intended use case at the start of the project was an algorithm that could reduce tech time and volume by correctly screening out true negatives, allowing human technologists to focus their time on more difficult, potentially positive cases. Furthermore, the clinical setting that this would be implemented in has very rigorous regulations and requirements. In our study, the model performance never reached a level where it was felt that an operational benefit would be realized from validating and implementing this algorithm into the clinical lab.

### Object detection

There are several key features of the object detection model that deserve extra discussion. First, true-positive detection mostly had high associated confidence scores (Fig. 2). Next, although tile borders have many obvious false positives, these predictions mostly have very low predicted confidence. Finally, most of the high-confidence false positives represent objects that are at least plausibly colored and shaped like AFB. Therefore, object detection model performance appears to be high when AFB are set against a clean, empty background, and most of the false-negative objects are adjacent to or embedded in background debris. The background matrix is also extremely important as sputum, which is our largest single category of specimens, often contains extensive background debris and non-*Mycobacteria* that may on occasion take up the stain. Notably, there were more false positives on sputum compared to the other sample types, which would dramatically increase the amount of manual slide reviews required by laboratory staff in clinical implementation. We suspect that distinguishing real AFB embedded in smear clutter from mimics or matrix debris (especially from sputum samples) is likely the biggest outstanding challenge for this task.

Although the model was trained for high-sensitivity detection to screen out negative smears and improve time to result reporting, there were at least four specimens that were negative by the model, but positive by manual AO. This discrepancy could be attributed to errors in sampling, non-homogenous specimens, and variations in smear preparation and staining.

Additionally, when assessing model accuracy, it must be noted that the training and validation sets for object detection were highly enriched for AFB-positive tiles and difficult negative tiles (those that overlapped with previous false positives). As the ratio of positive to negative training data was highly enriched for positive samples compared to the expected reality of clinical testing, it is possible that this may have reduced the generalizability of our model. Furthermore, as our validation set was specifically selected positive tiles and hard negative tiles, the precision, recall, and F1 scores would not be representative of object level performance on an unbiased sample of tiles, such as a new clinical slide.

### Scanner performance

During this study we collected objective parameters (scan time and output file size as a relation to scan area) between two leading scanners (Pramana HT2 and Hamamatsu S360) (Table S1). The average scan time for the Pramana HT-2 was 567 seconds and the average scan time for the Hamamatsu was 330 seconds. Additionally, the average file size from the Pramana HT-2 was 1.6 GB per slide compared to 8.3 GB for the Hamamatsu. This difference in file size was because the Hamamatsu output did not support compression of all Z-layers into one final image. Qualitatively, we found scans from the HT2 to be generally more useful than those from the S360, with fewer focus errors and better image quality. As a result, confidently distinguishing AFB from background when annotating HT2 scans was easier, and we chose to focus our efforts in validating our model on only the HT2 scanner. However, the increased scan time on the HT2 also reduced the operational viability of implementing an AI based tool in our clinical laboratory.

### Limitations and future directions

#### Annotations

Limitations of this study include paucity of annotations for AFB mimics and other non-AFB objects. Inclusion and explicit labeling of these “hard negative” samples can substantially reduce false positive prediction rates. Future work that includes more annotations of these mimics, especially on negative WSI might increase model performance. Additionally, tiles for labeling were created with an inconsistent approach due to the change in labeling and annotation software. In the future, a more consistent and/or algorithmic approach to tile generation could ensure better tiles are presented for labeling. Furthermore, there was no formal review process for human-created labels. Although all labels were created by highly trained and skilled laboratory staff, future work processes with a label verification step may improve quality of work and maintain consistency.

#### Size of training set

The size of our annotated training set, ~10,000 manual annotations over a few thousand tiles, is relatively small on the scale of modern machine learning data sets which may have millions of annotations and hundreds of thousands of images. However, annotating AFB on glass slides is extremely challenging and time-consuming and manually creating a larger data set may not be feasible for other groups. Future work, perhaps building off this data set, could explore the use of weakly-supervised learning approaches to increase the size and quality of the training data set. Furthermore, our training set was not large enough to spilt into different sample types, and future data sets that are large enough to support this may see performance gains.

#### Image registration

An additional limitation of our study is that we digitized the same glass slide with multiple scanners but did not use image registration to link positive annotations across multiple slides. This would help increase the data set diversity. Although we attempted to use image registration, the existing techniques we tested were geared towards histopathology and tissue sections and performance was extremely low on Kinyoun-stained smears. Also, as AFB are extremely small, image registration needs to be accurate to the micron – which is beyond the accuracy of most pre-existing techniques. Future work improving image registration will help create data sets that may yield better performance, including with different scanners.

#### Model training

Our approach to training the model had several specific limitations. With a pre-extracted tile data set, our ability to perform weakly supervised training was limited. In the future, an efficiently implemented WSI level data set would allow high confidence predictions to be cached between epochs, and supplemental new positives tiles based on high confidence predictions could be added to the training cycle dynamically per epoch. Furthermore, our choice of models was influenced largely by availability on torchvision; newer models such as DETR (30) would likely show improved performance on our data set.

For WSI level predictions, newer techniques such as multiple instance learning (MIL) (31), a weakly-supervised approach, could potentially outperform our simple density threshold decision rule. However, MIL can overfit badly, especially in a scenario such as this with comparatively few WSI, and MIL can struggle with extreme “needle-in-a-haystack” problems such as ours where, out of 10,000 tiles sampled from a 1 + WSI, there might be less than 10 positive tiles.

Strengths of this study include transparent release of our entire model, the focus on implementation as opposed to optimization, and perhaps most importantly, the creation of a publicly available data set of AFB organisms annotated by true experts in the field. While the model we developed is not yet strong enough to justify implementation in our laboratory, we are optimistic that our work and data set will help other groups tackle this difficult problem. The total GPU wattage draw for all training and testing of this model was approximately 570 kilowatt hours which, at the average US energy cost of 15 cents per kilowatt hour is <$100 spent on power consumption. With the size of our data set, further training and tuning of this model would be possible on moderately powerful commercial gaming hardware, hopefully allowing groups of all sizes and resource levels to benefit from this work.

## Data Availability

Code for this project as well as supplemental material is provided on GitHub at https://github.com/ARUP-RI/afb_detection. We used the commercial service comet.ml to iterate and track machine learning training runs. Additionally, the model and dataset are available at https://huggingface.co/arup-ri/afb.

## SUPPLEMENTAL MATERIAL

Supplemental materials spectrum.00602-25-s0001.docx Dataset split, scan comparison study, and representative random sampling of tiles from validation set.

ASM does not own the copyrights to Supplemental Material that may be linked to, or accessed through, an article. The authors have granted ASM a non-exclusive, world-wide license to publish the Supplemental Material files. Please contact the corresponding author directly for reuse.
