# Supplementary material for: Use of a convolutional neural network for direct detection of acid-fast bacilli from clinical specimens
Source: Microbiol Spectr. 2025 Jun 23;13(8):e00602-25. doi: 10.1128/spectrum.00602-25 (PMC12323672; doi:10.1128/spectrum.00602-25)
Supplement: Supplemental materials — Dataset split, scan comparison study, and representative random sampling of tiles from validation set. [file spectrum.00602-25-s0001.docx]

**Supplementary Material**

|  | Table S1: Scanner Settings and Performance Comparison | |  |
| --- | --- | --- | --- |
|  |  |  |  |
|  | **Scanner** | **Pramana HT-2** | **Hamamatsu 360** |
| **Settings*** | Magnification | 40x | 40x |
|  | Resolution (microns per pixel) | 0.25 | 0.23 |
|  | Z-Layers | 9 | 9 |
|  | Z-layer interval (um) | 1.25 | 1.3 |
| **Performance** | Average Scan Time (s) | 597 | 330.7 |
|  | Average Scan Area (mm^2^) | 323.4 | 311.9 |
|  | Average File Size (GB) | 1.6 | 8.3 |
|  | Scan time (s/mm^2^) | 1.8 | 1.1 |
|  | File Size (mb/mm^2^) | 4.9 | 26.5 |

**Table S2. Object detection and slide-classification dataset splits summary**

| Data Split | WSIs | Specimens | AFB+ Specimens | Annotated Tiles | Total Tiles | Unique Object Annotations |
| --- | --- | --- | --- | --- | --- | --- |
| 1^st^ round Object Training | 387 | 121 | 44 | 3683 | 15353 | 10964 |
| 1^st^ round Object Validation | 188 | 58 | 20 | 420 | 6330 | 447 |
| 2^nd^ round Object Training | 386 | 121 | 44 | 11414 | 48193 | 10964 |
| 2^nd^ round Object Validation | 188 | 58 | 20 | 408 | 5072 | 447 |
| WSI Validation | 188 | 111 | 32 | N/A | 1,880,000 | N/A |

**Figure S1 –** Representative random sampling of tiles from our validation set

**
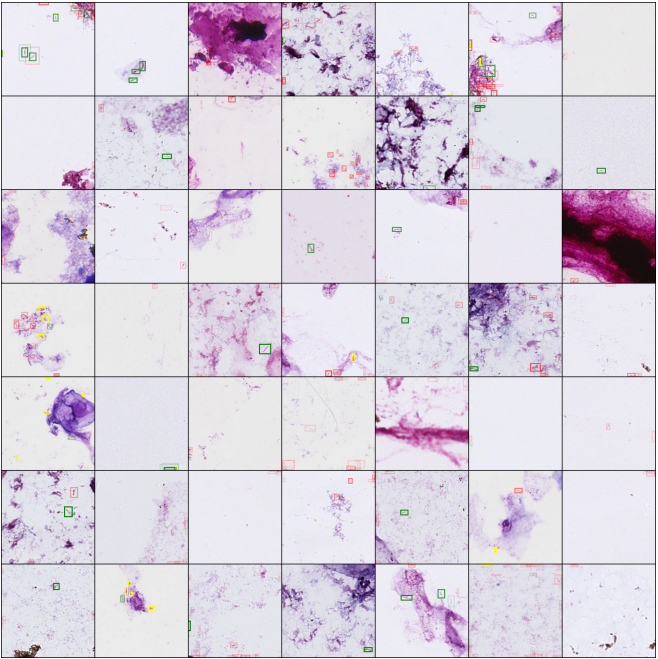
**
